# Differentiating tardive dyskinesia: a video-based review of antipsychotic-induced movement disorders in clinical practice

**DOI:** 10.1017/S109285292000200X

**Published:** 2020-11-20

**Authors:** Robert A. Hauser, Jonathan M. Meyer, Stewart A. Factor, Cynthia L. Comella, Caroline M. Tanner, Rose Mary Xavier, Stanley N. Caroff, Leslie Lundt

**Affiliations:** 1 Department of Neurology, University of South Florida Parkinson’s Disease and Movement Disorders Center, Tampa, Florida, USA; 2 Department of Psychiatry, University of California, San Diego, La Jolla, California, USA; 3 Department of Neurology, Emory University School of Medicine, Atlanta, Georgia, USA; 4 Department of Neurological Sciences, Rush University Medical Center, Chicago, Illinois, USA; 5 San Francisco Veterans Affairs Health Care System, Department of Neurology, University of California San Francisco, San Francisco, California, USA; 6 School of Nursing, The University of North Carolina at Chapel Hill, Chapel Hill, North Carolina, USA; 7 Perelman School of Medicine, University of Pennsylvania, Philadelphia, Pennsylvania, USA; 8 Department of Psychiatry, Corporal Michael J. Crescenz Veterans Affairs Medical Center, Philadelphia, Pennsylvania, USA; 9 Medical Affairs, Neurocrine Biosciences, Inc., San Diego, California, USA

**Keywords:** tardive dyskinesia, videos, drug-induced movement disorders, VMAT2 inhibitors, treatment

## Abstract

Accurate diagnosis and appropriate treatment of tardive dyskinesia (TD) are imperative, as its symptoms can be highly disruptive to both patients and their caregivers. Misdiagnosis can lead to incorrect interventions with suboptimal or even deleterious results. To aid in the identification and differentiation of TD in the psychiatric practice setting, we review its clinical features and movement phenomenology, as well as those of other antipsychotic-induced movement disorders, with accompanying links to illustrative videos. Exposure to dopamine receptor blocking agents (DRBAs) such as antipsychotics or antiemetics is associated with a spectrum of movement disorders including TD. The differential diagnosis of TD is based on history of DRBA exposure, recent discontinuation or dose reduction of a DRBA, and movement phenomenology. Common diagnostic challenges are the abnormal behaviors and dyskinesias associated with advanced age or chronic mental illness, and other movement disorders associated with DRBA therapy, such as akathisia, parkinsonian tremor, and tremor related to use of mood stabilizing agents (eg, lithium, divalproex). Duration of exposure may help rule out acute drug-induced syndromes such as acute dystonia or acute/subacute akathisia. Another important consideration is the potential for TD to present together with other drug-induced movement disorders (eg, parkinsonism, parkinsonian tremor, and postural tremor from mood stabilizers) in the same patient, which can complicate both diagnosis and management. After documentation of the phenomenology, severity, and distribution of TD movements, treatment options should be reviewed with the patient and caregivers.

## Introduction

Drug-induced movement disorders are caused by a variety of medications, including, but not limited to, antimicrobials, antiarrhythmics, antiemetics, antiepileptics, and psychotropic medications (eg, antidepressants, mood stabilizers, stimulants, and antipsychotics).^1–3^ The movements associated with these disorders are loosely categorized as hypokinetic, characterized by insufficient movement with reduced speed or magnitude (ie, parkinsonism), or hyperkinetic, characterized by excessive movement with increased velocity or amplitude (eg, chorea, dystonia, stereotypy, tremor). The most common drugs to cause movement disorders are dopamine receptor blocking agents (DRBAs) such as antipsychotics or antiemetics.^1^ Tardive dyskinesia (TD) is a persistent, irreversible DRBA-induced movement disorder with a broad phenotype that can include stereotypy, chorea, athetosis, dystonic movements, akathisia, or tic-like features. While these and other similar movements/movement disorders have recently been collectively referred to as tardive syndromes, for the purposes of this paper, we will focus on TD as a distinct movement disorder.

The pathophysiology of TD is not fully elucidated, but it is thought to be the result of upregulation and hypersensitivity of postsynaptic dopamine receptors.^4,5^ Until recently, there were no medications approved specifically for the treatment of TD. Treatment options were limited to dosing adjustments of the offending agents or off-label use of medications and herbal supplements with limited or no evidence of efficacy. The common use of anticholinergics often leads to exacerbation of choreiform movements of TD.^6^ In 2017, two vesicular monoamine transporter type 2 (VMAT2) inhibitors were introduced in the US, valbenazine, a valine ester of a highly selective isomeric metabolite of tetrabenazine ([+]-α-dihydrotetrabenazine), and deutetrabenazine, a deuterated form of tetrabenazine.^7,8^ Both drugs have demonstrated safety and efficacy in double-blind, placebo-controlled clinical trials and are considered first-line therapies for adults with TD.^6,9–12^

With the approval of new VMAT2 inhibitors came renewed clinical interest in improving the recognition and management of TD.^13–16^ Accurate diagnosis and appropriate treatment are imperative, as TD symptoms can be highly disruptive for both patients and their caregivers, causing embarrassment, isolation, behavioral disturbances, DRBA treatment non-adherence, impairment in daily functioning, and reduced quality of life.^14,17,18^ Misdiagnosis may lead to improper treatment (eg, TD being misdiagnosed as drug-induced parkinsonism and treated with an anticholinergic), with suboptimal or even deleterious results. Arriving at the correct diagnosis is complicated by the overlap of TD phenomenology with that of other DRBA-induced movement disorders, along with limited provider familiarity with the spectrum of movement disorders associated with TD and drugs that cause it. Another challenge in recognizing TD is the historic deemphasis on TD in training curricula, and the common categorization of all antipsychotic-induced movement disorders as “extrapyramidal symptoms” (EPS). The misuse of the term EPS can be harmful as TD is distinct from other DRBA-related movement disorders in pathophysiology, presentation, and treatment. Additionally, TD can present together with other drug-induced disorders (eg, parkinsonism, parkinsonian tremor, postural tremor from mood stabilizers) in the same patient. Finally, TD movements can be mistaken for abnormal behaviors associated with underlying psychiatric conditions, such as mannerisms, compulsions, catatonia, or spontaneous dyskinesias.^2,17,19^

The assessment of movement disorders often requires thorough consideration of a long list of primary and acquired etiologies. However, many of the disorders in the differential diagnosis of TD are rare and unlikely to be encountered in routine psychiatric practice. The most likely diagnostic challenges are the abnormal behaviors associated with age or chronic mental illness, as well as other movement disorders associated with DRBA therapy, such as parkinsonian tremor or postural tremor related to lithium or divalproex.^2,17,19^ Here, we review the clinical features and different movements associated with TD and other DRBA-induced movement disorders, with accompanying links to videos illustrating TD and non-TD movements, to aid in the identification and differentiation of TD in the psychiatric practice setting.

## Tardive DRBA-Induced Movement Disorders

### Tardive dyskinesia

#### Clinical course

As described in DSM-5, TD typically appears after at least a few months of DRBA use and may develop even sooner in older patients.^20^ However, there is no “safe” minimum period; TD can appear after weeks of DRBA exposure (Table 1).^19^ Onset of TD is variable and often insidious, evolving to a full syndrome rapidly or over time, with symptoms ranging in severity from “mild” to disabling or even life-threatening.^21^ This is followed by a chronic, but sometimes waxing and waning, course.^17^ Often, cessation or dose reduction of an inciting DRBA can trigger or exacerbate TD that had been “masked” by DRBA treatment.^17,22^ Conversely, increasing the DRBA dose can sometimes appear to ameliorate TD by “masking” its symptoms (Table 2).^5,17^

Once TD has become established, few patients will spontaneously remit even after cessation of the offending agent.^23–26^ Therefore, careful visual observation and questioning of the patient to identify early changes in motor behavior should be a standard part of the examination at every clinical visit. If TD is suspected, formal scales such as the Abnormal Involuntary Movement Scale (AIMS) and/or less structured overall patient assessments can be conducted to assess the extent, severity, and impact of the movements.^27^

#### Risk factors

Increasing numbers of patients are at risk for TD as the clinical use of DRBA medications, especially second-generation antipsychotic agents, expands beyond treatment of schizophrenia to include mood disorders such as bipolar disorder and refractory depression, as well as off-label use for agitation associated with dementia, and challenging behaviors in individuals with intellectual or developmental disabilities or other neurodevelopmental disorders.^28–32^ While second-generation antipsychotics are less likely to cause TD than first-generation antipsychotics, growing evidence indicates that the risk of developing TD is not insignificant for both second- and first-generation antipsychotics.^33–38^ A recent meta-analysis found that the prevalence of TD was 30% in patients receiving first-generation antipsychotics, 21% in patients receiving second-generation antipsychotics, and 7% in first-generation naïve patients who had only received second-generation antipsychotics.^34^

In addition to DRBA exposure, a recent targeted literature review suggested the following risk factors for TD: older age, female sex, white or African descent, longer illness duration, intellectual disability or brain damage, negative symptoms in schizophrenia, mood disorders, cognitive symptoms in mood disorders, genetic polymorphisms involving DRBA metabolism and dopamine function, diabetes, smoking, alcohol or substance abuse, intermittent DRBA treatment, and prior adverse reaction to DRBAs (eg, withdrawal-emergent dyskinesia, parkinsonism, akathisia, dystonia, or treatment-emergent dyskinesia).^39^ Among these, older age appears to be the greatest source of risk, with TD incidence rates fivefold greater than those among younger patients.^39^

#### Movement phenomenology

TD is characterized by persistent, hyperkinetic, involuntary movements of the mouth, jaw, tongue, face, trunk, or extremities, often in combination, that can cause embarrassment, impaired functioning, and behavioral disturbances, which can significantly impact quality of life for both patients and caregivers.^14,17,18^ TD movements are most often described as choreoathetotic: choreic (irregular, dance-like) and/or athetotic (slow, writhing) (Table 1). While both can occur throughout the body, the oral–buccal–lingual movements, such as irregular lip and jaw movements or tongue twisting, are considered the “classic” manifestations of TD [VIDEO 1].^17,19,40^ Involuntary movements in the oral–buccal–lingual region account for many TD cases (with some estimates reaching 60%–80%) and can lead to difficulty speaking, swallowing, and eating, lip and tongue biting, and the cracking or grinding down of teeth. Irregular movements of facial muscles may also occur such as increased blinking or blepharospasm. Chorea may also be present as “piano-playing” of the fingers/hands or irregular movement of the feet/toes [VIDEO 2]. In addition, choreoathetoid movements may occur in the neck, upper and lower extremities, and trunk [VIDEO 3]. In more extreme cases, leg or trunk involvement or excessive arm or truncal motion can affect ambulation [VIDEO 4]. Wild, flinging movements of the limbs and trunk are termed ballism, which is less common.

Stereotypies, or repetitive, purposeless movements that can be complex and derived from our “normal” repertoire of movements are also very common in TD. Examples of TD stereotypies include repetitive chewing movements or lip puckering, hand rubbing or wringing, pelvic thrusting, and truncal rocking [VIDEO 5 and VIDEO 6].

### Tardive dystonia

Dystonia can occur in an acute form that appears within hours or days of initiating or increasing DRBA dosage and resolves following discontinuation of the DRBA (*see acute dystonia*), but it also occurs in a tardive form.^2^ While tardive dystonia is classified in DSM-5 as a separate diagnosis from TD,^20^ dystonic-like movements may be a part of TD.^19^ Characteristic symptoms of tardive dystonia are involuntary, sustained muscle contractions that result in repetitive and sustained twisting or pulling movements and/or abnormal postures (Table 1). The contractions are usually focal and most often affect the neck, jaw, eyes, mouth, and face, causing torticollis, retrocollis, or anterocollis; blepharospasm; jaw opening or trismus; grimacing; or sustained tongue protrusion, dysphagia, or respiratory stridor [VIDEO 7].^2,41,42^ Less frequently, dystonia of the trunk or extremities can present as well. Associated subjective symptoms can include pain, anxiety, and distress. While dystonic movements may be repetitive, they differ from stereotypies in that they are less complex and do not appear to be part of the “normal” repertoire of movements. Dystonic movements are usually more sustained, causing abnormal postures that are typically patterned. Some patients with TD (ie, predominantly choreic and/or stereotypic movements) may also have dystonic movements, although to a lesser degree. It is important to differentiate whether tardive dystonia or TD (chorea or stereotypy) predominates, as there are different treatment options depending on the predominating movements.

### Tardive akathisia

Akathisia is characterized by subjective feelings of inner restlessness or tension and an urge to move, often manifested as an inability to remain seated (Table 1). Given akathisia can only be assessed in the context of these subjective complaints, a representative video was not included. Akathisia usually occurs in an acute/subacute form (*see acute/subacute akathisia*), with 90% of cases occurring within 90 days of initiating DRBA treatment, and complete resolution following treatment discontinuation; however, it can also occur in a late or tardive form that persists or worsens after DRBA discontinuation.^2^ The tardive form is often seen in combination with other tardive movement subtypes (eg, stereotypies, choreoathetoid movements, or dystonia), which can serve as a clue to the tardive character of the movements.^19,43^ Furthermore, this form of akathisia frequently persists for years and is difficult to treat.

### Tardive myoclonus, tics, and tremor

Rarely, DRBA-induced myoclonus, tics, and tremor can occur as tardive variants.^2,17,44^ Myoclonus usually presents as prominent postural, spontaneous, or stimulus-sensitive jerk-like movements in the upper extremities. Tics present as sudden, brief, stereotyped, involuntary or semivoluntary, semipurposeful movements which can be simple or complex (motor tics) or sounds (phonic tics) and are clinically indistinguishable from the tics of Tourette syndrome. These movements are associated with an urge that is diminished with completing the movement and are sometimes referred to as tardive tourettism. Tremor is characterized as a rhythmic, repetitive oscillation of a body part about a joint and is most often observed in the hands. Unlike DRBA-induced parkinsonian tremor which is usually a resting tremor (ie, occurs when the involved muscles are at rest), tardive tremor is usually action/postural (occurs while holding involved muscles in a static position against gravity).^17,19,45^ In addition, tardive tremor is rare compared to parkinsonian tremor. Tardive tremor can only be diagnosed when other agents that induce postural tremor (eg, lithium, valproate) have been removed, and the tremor is demonstrated to behave in a manner consistent with a tardive condition (ie, worsens with reduction or discontinuation of DRBA and/or improves with an increase in DRBA dosage or treatment with a VMAT2 inhibitor).

## Acute/Subacute DRBA-Induced Movement Disorders

DRBAs are associated with a number of acute/subacute movement disorders, including parkinsonism/parkinsonian tremor, acute dystonia, acute or subacute akathisia, and the rare but potentially fatal neuroleptic malignant syndrome (NMS). These were recently classified by Factor et al. as disorders that are subacute in onset and chronic but reversible (DRBA-induced parkinsonism/parkinsonian tremor and acute/subacute akathisia) vs those disorders that are acute in onset and reversible (acute dystonia, NMS).^1^ Clinical characteristics of these disorders and how they can be differentiated from TD are described in the following sections.

### Parkinsonism/parkinsonian tremor

Signs of DRBA-induced parkinsonism mimic those of the motor syndrome of Parkinson’s disease (Table 1), with prevalence rates ranging from 15% to 40% of DRBA-treated patients.^2,43^ Typical onset of parkinsonism is within days to weeks of starting a DRBA or increasing the dosage, with 50% to 75% of cases occurring within the first month and 90% occurring within the first 3 months.^2,41,43^ However, onset can sometimes occur years after initiating or increasing DRBA dosage. The reason for this is unclear. The most common signs of parkinsonism are bradykinesia (slowing of speed and decreased amplitude of movements such as finger tapping) [VIDEO 8] and tremor (a rhythmic, patterned oscillation of a body part about a joint).^46^ Notably, some patients may exhibit parkinsonian tremor without obvious bradykinesia or bradykinesia without tremor. The classic parkinsonian tremor is a resting tremor that is maximal at rest and abates or disappears with use of the involved muscles [VIDEO 9]. However, it may also be apparent with the arms outstretched (in a position of postural maintenance) or during action/movement. A frequency of 3 to 4 Hz is typical. It is most often observed in one or both hands as a “pill-rolling” motion or flexion/extension at the wrist. An asymmetric presentation of tremor (occurrence on one side) does not exclude the diagnosis of drug-induced tremor. Tremor can be detected in other areas besides the hands and occasionally affects perioral muscles, jaw, or tongue (see discussion below of rabbit syndrome) [VIDEO 10]. The rhythmic, repetitive movements of parkinsonian perioral tremor are quite different from the complex, stereotyped, and irregular orofacial movements of TD. Other manifestations of parkinsonism may include hypomimia (decreased facial expression), decreased blink rate, and reduced arm swing with walking, flexed posture, and shuffling or freezing gait. In addition, parkinsonism can include rigidity (stiffness) of the neck, trunk, and extremities. Parkinsonian signs are typically bilateral and usually symmetrical. Patients may also complain of fatigue or weakness.^47^

Risk factors for DRBA-induced parkinsonism include older age, female sex, brain structural abnormalities (eg, dementia, human immunodeficiency virus infection), family history of Parkinson’s disease, and previous adverse reaction to DRBAs.^2,41,48,49^ Although the risk of parkinsonism also correlates with increased DRBA dosages and potency, the dose–response relationship is often confounded by differences in susceptibility (eg, age of the population under study).^2^ Second-generation antipsychotics may have a lower risk of parkinsonism than high-potency first-generation antipsychotics such as haloperidol; however, even second-generation antipsychotics may cause significant parkinsonism in susceptible individuals.^2,50–52^

In contrast to TD, drug-induced parkinsonism and parkinsonian tremor represent a low postsynaptic dopamine state due to excessive dopamine receptor blockade in the striatum; therefore, it will generally improve with a cessation or reduction in DRBA dose and worsen with an increase in DRBA dose. Unlike TD, symptoms are generally reversible, in most cases within weeks or months of discontinuing DRBA treatment; however, in approximately 15% of cases (or up to 40% of cases in older patients), symptoms may persist after DRBA discontinuation or modification, raising the possibility of underlying Parkinson’s disease.^2,48,53^ Imaging the density of dopamine transporters with ^123^ioflupane (DaTscan) may be useful to distinguish drug-induced parkinsonism from Parkinson’s disease.^54^ Patients with drug-induced parkinsonism should have a normal amount of dopamine transporters, whereas those with Parkinson’s disease will have reductions. In cases where discontinuation or reducing dose or switching potency of DRBA treatment are not viable options, anticholinergic drugs (eg, benztropine, approved as adjunctive therapy for parkinsonism and may be useful for acute DRBA-induced dystonia^55^) or amantadine (approved for parkinsonism^56^) are often used; however, there is limited evidence from controlled trials supporting the use of these 3 agents (Table 2).^1^ Moreover, anticholinergics are associated with both peripheral and central nervous system adverse effects.^57^ The role of levodopa is unclear but may be contraindicated due to increased risk of psychotic symptoms. Paradoxically, anticholinergics can worsen choreatic TD symptoms as is true with levodopa; thus, it is important to distinguish between TD and parkinsonism/parkinsonian tremor before initiating anticholinergic treatment. As the only hyperkinetic feature of parkinsonism, tremor is sometimes confused with TD.^2,41^ However, the movements of parkinsonian tremor are distinct from those that are most often seen in TD: the dance-like, irregular “piano-playing” movements of chorea, the pulling movements of dystonia, and the repetitive but complex and purposeless movements of stereotypies. Rabbit syndrome represents a focal manifestation of parkinsonian tremor that is sometimes confused with TD, as the tremor primarily affects the jaw and can include rhythmic movements in the tongue or soft palate. The key to distinguishing this from TD is the regularity of the movement, despite the involvement of oral structures. As with other forms of parkinsonism, rabbit syndrome also responds to DRBA dose reduction.

### Acute dystonia/acute dystonic reactions

Onset of acute dystonia (or acute dystonic reaction) often occurs within hours of the first dose of a DRBA, presumably due to an acute low postsynaptic dopamine state, with 95% of cases appearing within the first 5 days of initiating or increasing dosage.^2^ Phenomenologically, acute dystonia is similar to the presentation of tardive dystonia described above, characterized by sustained muscle contractions causing twisting or pulling movements or abnormal postures of the head, neck, jaw, mouth, face, and eyes (eg, oculogyric crisis) (Table 1).^2,41,42^ If pharyngeal or laryngeal muscles are involved, this can pose a life-threatening emergency. Less frequently, dystonia of the trunk or extremities can present. Dystonic reactions can last a few seconds or several hours and may be sustained, fluctuating, or episodic.^47^ Symptoms of acute dystonia usually resolve within 24 to 48 hours of discontinuation of oral DRBAs.

Risk factors for acute dystonia include younger age (children and young adults are highly vulnerable), male sex, mood disorders, previous dystonic reactions, family history of dystonia, drug use, hypocalcemia, hypoparathyroidism, hyperthyroidism, and dehydration.^2,42,58,59^ Higher dosage and potency of DRBAs, and increased rate of titration, have been associated with higher risk of dystonia.^2,42,58,59^ Lower potency first-generation antipsychotics with weak dopamine antagonism and prominent anticholinergic (antimuscarinic) effects may have a lower risk of dystonia (eg, chlorpromazine), and second-generation antipsychotics also have lower dystonia risk.^2,52,60,61^ Patients who develop acute dystonia on one DRBA may be at risk for recurrence on others.

The movement phenomenologies of the acute and tardive forms of dystonia are difficult to distinguish; thus, acute dystonia is primarily distinguished from tardive dystonia by a rapid onset after DRBA administration and rapid resolution after cessation of DRBAs or rescue treatment with anticholinergics such as benztropine or intravenous diphenhydramine (approved for allergic reactions, anaphylaxis, motion sickness, and parkinsonism, but not for dystonia^62^).^2^

### Acute/subacute akathisia

Acute/subacute akathisia is estimated to occur in 20% to 35% of patients receiving a DRBA.^2,63^ Onset of symptoms may be within several days of initiating DRBA treatment or an increase in dose, and the incidence tends to increase with duration of DRBA treatment, with 50% of cases occurring within the first month and 90% occurring within the first 3 months.^2,41,64^ Symptoms usually resolve after DRBA discontinuation in acute/subacute akathisia but could worsen or persist in tardive akathisia. The symptoms of tardive akathisia and acute/subacute akathisia can be identical and are characterized by subjective feelings of inner restlessness or tension, and an urge to move, often with an inability to remain seated (Table 1). As with tardive akathisia, a representative video of acute akathisia was not included given that it can only be assessed in the context of these subjective complaints. Symptoms improve in some patients treated with propranolol, clonazepam, or serotonin 2A antagonists such as mirtazapine, although evidence for efficacy is limited and none of these treatments have been approved for akathisia.^17,65^ Critical evaluation of available evidence does not support the use of anticholinergics for akathisia.^65^

Risk factors for acute akathisia include increasing age, female sex, mood disorders, parkinsonism, negative symptoms in schizophrenia, cognitive impairment, iron deficiency, and prior akathisia.^2,47,66,67^ Second-generation antipsychotics have been associated with a significantly lower incidence of akathisia compared with first-generation antipsychotics in some studies.^2,63^

Acute/subacute akathisia may be difficult to distinguish from tardive akathisia from a phenomenological standpoint, since it can occur essentially at any time during DRBA treatment; however, early onset of akathisia after initiation or dose increase of a DRBA indicates a greater likelihood of response to discontinuing or decreasing dose, while longer duration of DRBA exposure increases the likelihood of tardive akathisia. In addition, tardive akathisia is quite uncommon and may be associated with more objective features of hyperactivity and less subjective discomfort than in the acute form.^68,69^

### Neuroleptic malignant syndrome

NMS is an extremely rare but potentially lethal (~5% if treated, 20% if untreated) reaction to DRBAs.^2,70^ Symptoms develop acutely, within hours to days, with two-thirds of cases occurring within 1 to 2 weeks after DRBA initiation or dose increase.^2^ NMS usually presents with hyperthermia, generalized severe “lead pipe” muscle rigidity with or without tremors, altered mental state (eg, confusion, delirium, stupor, or coma), and autonomic instability (Table 1).^2,71^ Other motor symptoms can include various dyskinesias, dystonia, myoclonus, dysarthria, dysphagia, and in its most extreme form, hypermetabolic crisis.^1^

Potential risk factors for developing NMS include dehydration, exhaustion, agitation, catatonia, prior episodes of NMS, rapid escalation of DRBA dose, use of multiple DRBAs, concurrent dopamine-depleting agents, lithium, and selective serotonin or serotonin-norepinephrine reuptake inhibitors (SSRIs or SNRIs).^2,72,73^ While NMS has been associated with both first-and second-generation antipsychotics, the risk is greater with high-potency first-generation antipsychotics, especially when given parenterally at high dosages and at a rapid rate. Haloperidol has been associated with about half of reported cases.^2^

Although acute dyskinesias can herald the onset of NMS, the rapid and acute onset of NMS, along with the development of systemic signs, distinguish it from the more indolent and localized movements of TD. DRBA treatment must be discontinued for NMS symptoms to resolve, and supportive treatments, including fluid management and temperature lowering interventions, immediately commenced. NMS is similar to other *acute/subacute* DRBA-induced movement disorders, in that VMAT2 inhibitors are likely to worsen symptoms, and they should be discontinued if NMS develops.^74^ When promptly diagnosed, symptoms most often resolve within 1 to 2 weeks after discontinuation of oral DRBAs.^2,72^ Some patients may benefit from supplemental treatment with benzodiazepines, dopaminergic drugs, muscle relaxants (eg, dantrolene), or even electroconvulsive therapy, although comparative controlled data are lacking and these agents are not approved to treat NMS.^2^

## Other Psychotropic Drug-Induced Movement Disorders

It is worth noting that other types of psychotropic medications, such as SSRIs, mood stabilizers, tricyclic antidepressants (TCAs), and stimulants, are routinely associated with movement disorders; however, none of these are persistent and all will resolve after drug discontinuation.^46,75–77^ Among these agents, the most commonly encountered movements are from exposure to mood stabilizers such as lithium and valproate. While these two medications are not commonly associated with parkinsonism, their use is frequently associated with a low amplitude, high frequency postural, or resting tremor.^46,75,78–80^ The tremor mainly affects the hands and is usually mild but can cause disability in some patients.^80^ Approximately, 20% of patients receiving an SSRI develop a mild hand tremor that resembles essential tremor, is typically postural/action in nature, emerges within 1 to 2 months of treatment initiation, and may respond to propranolol.^46,81^ SSRIs have also been reported to cause numerous other movement disorders, including akathisia, and dystonia but tremor is most commonly encountered.^46,75,82^ SSRI-induced tremor can also be an early manifestation of serotonin syndrome, or serotonin toxicity, which can occur following a dosage increase or addition of a concomitant serotonergic agonist. Onset of serotonin syndrome is usually rapid, often occurring within minutes of elevated serotonin activity, and symptoms (tremor, myoclonus, hyperreflexia, mental status changes, hyperthermia, and autonomic instability) can range from mild to life-threatening.^83^ Serotonin syndrome results from toxicity associated with use of a wide variety of serotonergic agents, including antidepressants, triptans, herbal products, and others and may be difficult to differentiate from NMS as they share core features.^1^ Severe episodes of serotonin syndrome are especially associated with drugs possessing monoamine oxidase–inhibiting properties (certain antidepressants and antibiotics [linezolid], methylene blue, St. John’s wort) and can be indistinguishable from NMS. Resolution of serotonin syndrome usually occurs rapidly after discontinuation of offending agents, although serotonin 2A receptor antagonists (eg, cyproheptadine) have been employed empirically in some cases. TCAs have been associated with myoclonus, akathisia, dystonia, and postural/action tremor of the hands and rarely with orofacial dyskinesia.^46,75,84^ The tremor, similar to that caused by other drugs listed above, can be disabling but is usually mild and can improve over time in some patients who remain on therapy.^46,85^ Finally, stimulants (eg, amphetamine, methylphenidate, and pemoline) have been reported to produce a variety of movement disorders such as dystonia, stereotypies, and tics.^75^

## Differential Diagnosis and Management of TD

The differential diagnosis of TD is based on a history (or suspected history) of DRBA exposure and its characteristic movement phenomenology in the absence of associated neurologic or cognitive defects or systemic signs of illness. There are no biomarkers for TD diagnosis or predictors of outcomes. Most importantly for clinicians, some of the movements of TD and many of its associated risk factors (eg, advanced age, mood disorder, diabetes, cognitive impairment, alcohol or drug abuse, and prior adverse reaction to DRBAs) overlap with those of other acute and subacute DRBA-induced movement disorders.

An important first step in the assessment for TD is careful visual observation and questioning of the patient and caregiver to identify the presence and character of any abnormal movements. Visual observation of changes in motor behavior should be a standard part of the mental status examination at every clinical visit. If TD is suspected, an assessment of the extent, severity, and impact of the movements should be conducted using the AIMS and/or less structured overall patient assessments.^27^ TD symptoms usually appear after at least a few months of DRBA use, but some patients may experience symptoms earlier;^20^ thus any patient taking a DRBA should be considered at risk for developing TD.^19^ Not all patients will report their abnormal movements, possibly because of embarrassment or greater concern about other psychiatric or medical conditions. Some individuals may not be aware of their abnormal movements due to deficits in cognition or insight due to underlying chronic schizophrenia.^14,86,87^ In these cases, caregivers and/or family members are helpful in providing information.

If DRBA treatment is reported or suspected, perhaps the most important differential for clinicians is distinguishing TD from the more acute or subacute forms of DRBA-induced movement disorders because the treatment approaches are very different. For example, parkinsonism/parkinsonian tremor, acute akathisia, and acute dystonia improve with DRBA reduction/discontinuation, whereas TD is likely to persist or temporarily worsen with these interventions, especially if TD is established and chronic. Likewise, anticholinergics will also worsen TD but improve parkinsonism. Conversely, VMAT2 inhibitors will improve TD symptoms but are likely to worsen parkinsonism/parkinsonian tremor and potentially other acute DRBA-induced movement disorders.^13^ It is also absolutely critical to distinguish parkinsonian tremor from the abnormal movements of TD. Parkinsonian tremor can worsen with introduction of a VMAT2 inhibitor and improve with reduction of the DRBA medication or use of an anticholinergic.

Acute or subacute syndromes such as acute dystonia, akathisia, parkinsonism/parkinsonian tremor, or NMS generally present within hours to weeks of initiating or increasing DRBA treatment, while TD is usually associated with more prolonged exposure; however, it is important to note that these distinctions are not absolute. As a rule of thumb, acute dystonia presents within hours to days of initiating DRBA therapy, whereas tardive dystonia presents after weeks to months of DRBA exposure. Akathisia can occur essentially at any time during DRBA treatment, although early onset after DRBA initiation or dose increase is more often associated with acute akathisia, which, in turn, responds to decreasing or discontinuing the DRBA.

Note that multiple movement disorders may be present in the same patient, especially elderly patients as they are at increased risk for TD and drug-induced parkinsonism. It is not uncommon for a patient on a DRBA to exhibit signs of TD (chorea/stereotypy) or tardive dystonia while also exhibiting parkinsonism (bradykinesia, decreased facial expression, small strides, and decreased arm swing) and/or parkinsonian tremor [VIDEO 11]. Management of these patients is complex in that VMAT2 inhibitors for TD may negatively impact parkinsonism and parkinsonian tremor, while reducing the DRBA to improve parkinsonism and parkinsonian tremor may exacerbate (“unmask”) TD movements. Similarly, anticholinergics prescribed for parkinsonian tremor can worsen choreiform movements of TD.

After documentation of severity, distribution, and phenomenology of TD, and laboratory investigation or neurological consultation (if necessary), treatment options should be reviewed with the patient and caregivers. In 2018, the authors of the American Academy of Neurology guidelines for tardive syndromes published updated recommendations that included VMAT2 inhibitors (valbenazine and deutetrabenazine), the first approved TD treatments with Level A evidence from well-designed clinical trial data.^6^ Based on this evidence, treatment with a VMAT2 inhibitor should be considered as first-line treatment for TD, regardless of whether or not the patient remains on DRBA treatment. DRBA treatment should be reviewed for possible switch or discontinuation (only in patients who are not psychotic and can be safely tapered) especially if TD is detected early;^23,24^ unfortunately, early signs of TD are often missed, and it may become irreversible once established. Moreover, supportive evidence of the impact of DRBA modification on the course of TD is limited (Level U evidence).^2,6,25^ There is very limited evidence (Level B or C) supporting other unapproved treatments such as clonazepam, Gingko biloba, and amantadine.^6^ Anticholinergics (eg, benztropine) may exacerbate TD and should never be initiated solely to treat TD. Unless significant parkinsonism or tardive dystonia are present, anticholinergics should be cautiously discontinued over a period of 2 to 4 weeks while monitoring for any possible worsening of parkinsonism or other anticholinergic discontinuation-related effects.^2,17,88^

In cases of TD in which other movement types (eg, tardive dystonia and tardive akathisia) predominate, the evidence on their pharmacological response to VMAT2 inhibitors, DRBA dose/type adjustment, etc., is limited^89–91^. However, the response of tardive dystonia and tardive akathisia to DRBAs may be similar to that of TD in that increasing DRBA dosages may temporarily suppress movements, reducing/discontinuing DRBAs may temporarily worsen movements while most chronic movements will persist. It should be noted that tardive dystonia may also respond to anticholinergics; clozapine may be particularly effective in some cases if an antipsychotic is indicated, and injections of botulinum toxin may be helpful in alleviating focal dystonic symptoms.

## Conclusions

Accurate diagnosis and appropriate treatment of TD are imperative, as the symptoms can be highly disruptive for both patients and their caregivers, causing impaired functioning and reduced quality of life. The differential diagnosis of TD is based on a history of DRBA exposure, duration of exposure, and movement phenomenology, as well as any history of associated systemic or neurological disease. An important first step is to identify the presence of any abnormal movements by developing a standard procedure for regular TD screening of all patients with exposure to DRBAs through careful visual observation and questioning of the patient and caregiver. The videos included in this review serve to illustrate the phenomenologic manifestations of TD, as well as those of other movement disorders that can be misdiagnosed as TD.

After documentation of TD severity, distribution, and phenomenology, treatment options should be reviewed with the patient and caregivers, including initiation of treatment with a VMAT2 inhibitor, and modification or discontinuation of anticholinergics and DRBAs if possible, based on psychiatric and other clinical considerations.

## Supplementary Material

videos **VIDEO 1.** Tardive dyskinesia. “Classic” oral–buccal–lingual chorea consisting of repetitive, irregular movements of mouth, lips, and tongue, along with increased blinking (blepharospasm), and truncal chorea.
**VIDEO 2.** Tardive dyskinesia. Chorea consisting of repetitive, irregular, dance-like movements of the fingers (“piano-playing”) and toes.
**VIDEO 3.** Tardive dyskinesia. Choreoathetotic (repetitive, irregular, writhing) movements of the trunk, legs, neck, hands, and fingers.
**VIDEO 4.** Tardive dyskinesia. High-amplitude chorea of the trunk, neck, and extremities.
**VIDEO 5.** Tardive dyskinesia. Stereotypies (repetitive, purposeless movements): lip puckering and inversion and truncal rocking.
**VIDEO 6.** Tardive dyskinesia. Stereotypies (repetitive, purposeless movements) of mouth/jaw/tongue including swallowing, puffing, and tongue thrusting.
**VIDEO 7.** Tardive dystonia. Retrocollis: repetitive, patterned, neck extension.
**VIDEO 8.** Parkinsonism. Bradykinesia: slow speed and loss of amplitude in finger tapping and marked decrease in facial expression (masking).
**VIDEO 9.** Parkinsonism. Parkinsonian tremor: rhythmic, 3 to 4 Hz resting tremor.
**VIDEO 10.** Parkinsonism. Parkinsonian tremor: rhythmic, 3 to 4 Hz tremor in lower lip and chin.
**VIDEO 11.** Tardive dyskinesia and parkinsonism. Oral–buccal–lingual movements and head rocking (tardive dyskinesia) and resting left-hand tremor (parkinsonian tremor).

## Figures and Tables

**Table 1.** Clinical Characteristics of Dopamine Receptor Blocking Agent (DRBA)-induced Movement Disorders

Characteristic	Tardive Dyskinesia	Dystonia		Akathisia		Parkinsonism	Neuroleptic Malignant Syndrome
		Acute	Tardive	Acute	Tardive		

Typical time to onset^a^	Weeks to years^b^	Hours to days	Weeks to years	Days to months	Weeks to years	Days or weeks to years	Hours to weeks

Movement phenomenology	Choreoathetotic (irregular, dance-like), athetotic (slow, writhing), and/or stereotypic (repetitive, purposeless) movements of the mouth, jaw, tongue, and face (mouth/jaw chewing, tongue protrusion, grimacing, lip smacking or pursing, blepharospasm); also, choreoathetotic and/or stereotypic movements of neck, trunk, and extremities (pianoplaying finger/hand movements, foot tapping, truncal rocking or thrusting)	Pulling, twisting, sustained, and repetitive movements or postures that are usually focal, involving the head, neck, eyes, mouth, jaw, tongue, and face (torticollis, trismus, jaw opening, grimacing, blepharospasm or oculogyric crisis, tongue protrusion, biting, or twisting)		Inner feeling of restlessness with urge to move and inability to maintain seated; may be associated with stereotypies such as foot tapping, shuffling, shifting weight, or rocking		Tremor and/or bradykinesia; also, rigidity of neck, trunk, and extremities, hypomimia, reduced blink rate, reduced arm swing, flexed posture, and shuffling or freezing gait; also, rabbit syndrome (a parkinsonian variant that includes jaw tremor)	Generalized “lead-pipe” rigidity with tremor (less frequently: various dyskinesias, dystonia, or myoclonus)
		
Other clinical features	Difficulty speaking, eating, or ambulating, embarrassment, social isolation	Muscle pain or cramps, distress, anxiety, dysarthria, dysphagia, respiratory stridor				Soft speech, dysphagia, fatigue	Hyperthermia, altered consciousness, autonomic instability, dysarthria, dysphagia (in extreme form: hypermetabolic crisis)

a Following DRBA initiation or change in dose. Onset may occur earlier or later than the typical time frames listed (see manuscript text for further information).

b TD may be “masked” by DRBA treatment and first appear after DRBAs are withdrawn.

**Table 2.** Key Differences in Pharmacologic Effects of Common Treatments on Dopamine Receptor Blocking Agent (DRBA)-lnduced Movement Disorders

Action	Tardive Dyskinesia	Dystonia		Akathisia		Parkinsonism	Neuroleptic Malignant Syndrome
		Acute	Tardive	Acute	Tardive		

Increase DRBA dose	May initially “mask” symptoms^a^	May trigger or worsen	May initially “mask” symptoms	May trigger or worsen	May initially “mask” symptoms	May trigger or worsen	Must discontinue DRBA for recovery

Discontinue DRBA or reduce dose	Generally no effect, but may improve over time in some patients^b^	Improves	Generally no effect, but may improve over time in some patients^c^	Improves	Generally no effect, but may improve over time in some patients^c^	Improves	

Add VMAT2 inhibitor	Improves (approved for treatment of TD)^d^	May worsen	May improve	Insufficient data	Insufficient data	May worsen	Must discontinue VMAT2 inhibitors

Add anticholinergic	May worsen	May improve^e^	May improve^e^	Insufficient data	Insufficient data	Improves (approved for treatment of parkinsonism)^e^	Must discontinue anticholinergic

Discontinue anticholinergic	May improve	May worsen	May worsen	Insufficient data	Insufficient data	May worsen

Abbreviations: TD, tardive dyskinesia; VMAT2, vesicular monoamine transporter type 2.

a Increasing DRBA dose may diminish chances of recovery from TD.

b DRBA discontinuation or reduction can initially trigger or exacerbate TD that had been “masked” by DRBA treatment.

c Limited data available.

d Valbenazine and deutetrabenazine are approved in the US for the treatment of TD in adults and are recommended as first-line treatment for TD.

e Benztropine is approved in the US for all forms of parkinsonism and may be useful for acute DRBA-induced dystonia. Anticholinergics can aggravate TD and should not be used for TD.
